# The impact of anthropogenic transformation of urban soils on ectomycorrhizal fungal communities associated with silver birch (*Betula pendula* Roth.) growth in natural versus urban soils

**DOI:** 10.1038/s41598-023-48592-6

**Published:** 2023-12-02

**Authors:** Jacek Olchowik, Paweł Jankowski, Marzena Suchocka, Tadeusz Malewski, Adam Wiesiołek, Dorota Hilszczańska

**Affiliations:** 1 Department of Plant Protection, Institute of Horticultural Sciences, Warsaw University of Life Sciences-SGGW, Warsaw, Poland; 2 Department of Computer Information Systems, Institute of Information Technology, Warsaw University of Life Sciences-SGGW, Warsaw, Poland; 3 Department of Landscape Architecture, Institute of Environmental Engineering, Warsaw University of Life Sciences-SGGW, Warsaw, Poland; 4 Department of Molecular and Biometric Techniques, Museum and Institute of Zoology, Polish Academy of Science, Warsaw, Poland; 5 Department of Forest Ecology, Forest Research Institute, Sękocin Stary, Poland

**Keywords:** Ecology, Plant sciences

## Abstract

*Betula pendula* Roth. is considered a pioneering plant species important for urban ecosystems. Based on the sequencing of fungal ITS, we characterized the ectomycorrhizal (ECM) communities of twenty silver birch trees growing in a contaminated, highly anthropo-pressured urban environment and in a natural reserve site. We analysed chemical properties of each tree soil samples, focusing on effects of anthropogenic transformation. Three effects of urbanization: high heavy metal content, increased salinity and soil alkalinity, were highly correlated. The examined trees were divided into two forest and two urban clusters according to the level of anthropogenic soil change. The effect of soil transformation on the ECM communities was studied, with the assumption that stronger urban transformation leads to lower ECM vitality and diversity. The results of the study did not confirm the above hypothesis. The ECM colonization was above 80% in all clusters, but the forest clusters had significantly higher share of vital non-ECM root tips than the urban ones. Eleven mycorrhizal fungal species were identified varying from seven to nine and with seven species observed in the most contaminated urban plot. However, the lowest Shannon species diversity index was found in the most natural forest cluster. In conclusion, our findings demonstrate no significant negative effect of the urban stresses on the ECM communities of silver birch suggesting that both forest and urban trees have the potential to generate a similar set of ECM taxa.

## Main

In contrast to non-urban soils, urban soils have a significantly higher pH, which results mainly from the high concentration of artificial materials, especially construction waste which is often very alkaline due to its large limestone content^1–3^. Moreover, the urban soils in northern countries have a higher salt content^3^ which results from the use of the de-icing salts during the cold winters^4,5^. Finally, the urban soils have higher concentrations of pollutants, including heavy metals such as Cd, Pb, Zn and Cu^3,6–8^. The sources of high pollutants accumulation are also anthropogenic: road traffic with petrol combustion, industrial emissions, weathering of building and pavement surface but also residential, commercial, recreational, and even agricultural land use^6,9,10^.

Apart from threats to plant development resulting from the difference in the chemical composition of urban soils, urban trees face numerous stress factors, like limited root space^11,12^ or heat island effect^13^.

Silver birches (*Betula pendula* Roth.) occur naturally throughout most of Europe up to central Siberia. It is one of the most important deciduous species in Polish forests, accounting for 7.3% of Polish forest coverage^14^. *B. pendula* grows best in acidic soils. Furthermore, silver birches are widely planted in urban areas, roadsides, and parkland because of its tolerance to a broad range of site conditions, and poor soils, birch is a pioneering tree often used for land reclamation and revegetation^15^. This species shows a high resilience to toxic soil conditions, even those heavily contaminated with metals^16–19^.

The roots of silver birch are associated with a large number of ectomycorrhizal (ECM) fungi^20^. Current evidence suggests that the ECM fungi communities may play an important role in host nutrition with organic nitrogen and in the storage of carbon by the plant in addition to improving overall nutrient uptake of the host tree^21–24^. It is also evident that ECM can offer transport of water via its hyphal network^25,26^. Finally, ectomycorrhizal fungi may limit the uptake of heavy metals^27,28^.

*B. pendula* is considered a resistant tree species, used in reclaimed areas. It is considered a bioindicator of heavy metal pollution in urban biotopes^1,29,30^ and an indicator species for the environmental sites^31–33^. A wide range of physiological, ecological and biological characteristics of *B. pendula* in the conditions of urban environment have been studied in detail. The recent research^34,35^, showed high changes between many tree features compared between the control values and values found in the urban habitat included: seed germination, curved leaf apex, crown volume, pollen viability, the amount of abnormal pollen, vital state of trees, seed quality, crown area, stomata area, thickness of the lower epidermis, stomata density on leaf blade, seed productivity, pollen fertility, width of fruit (female) catkins, number of scales in fruit (female) catkins, thickness of palisade parenchyma, tree height, stomata length, pollen tube length and trunk diameter. Authors have not found any study concerning the effect of urban environment on the ECM communities of the *B. pendula* trees.

Several studies have investigated the ECM communities occurring in urban sites e.g.^11,12,36–44^. However, none of these studies have compared the ECM species associated with the *B. pendula*. Therefore, the main goal of this study was to characterise and compare the ECM communities of *B. pendula* growing in two sites in the Masovian Voivodeship in central-eastern Poland. The first, urban site, was chosen in a contaminated post-industrial and densely populated district of Warsaw, Ursus, developed around the currently closed tractor factory. The second, control site, was located in the John III Sobieski Nature Reserve, protected since 1952. The additional aim of the study was to analyse the possible effects of soil chemical properties, focusing on heavy metal content, salinity, and soil pH, on the ECM communities. Accordingly, the study hypothesis was that due to more challenging conditions and additional stresses, the urban ECM communities of silver birch are less developed than the forest ones, with lower vitality and lower diversity of species.

## Results

### The relation between the chemical soil and ECM characteristics of individual trees

The presented study is based on analysis of twenty soil samples, each related to a different tree growing either in forest (ten trees) or urban (ten trees) localizations. In the first step, the relations between the chemical and ECM characteristics of the individual soil samples were examined, using the Spearman correlations.

**Figure 1:**
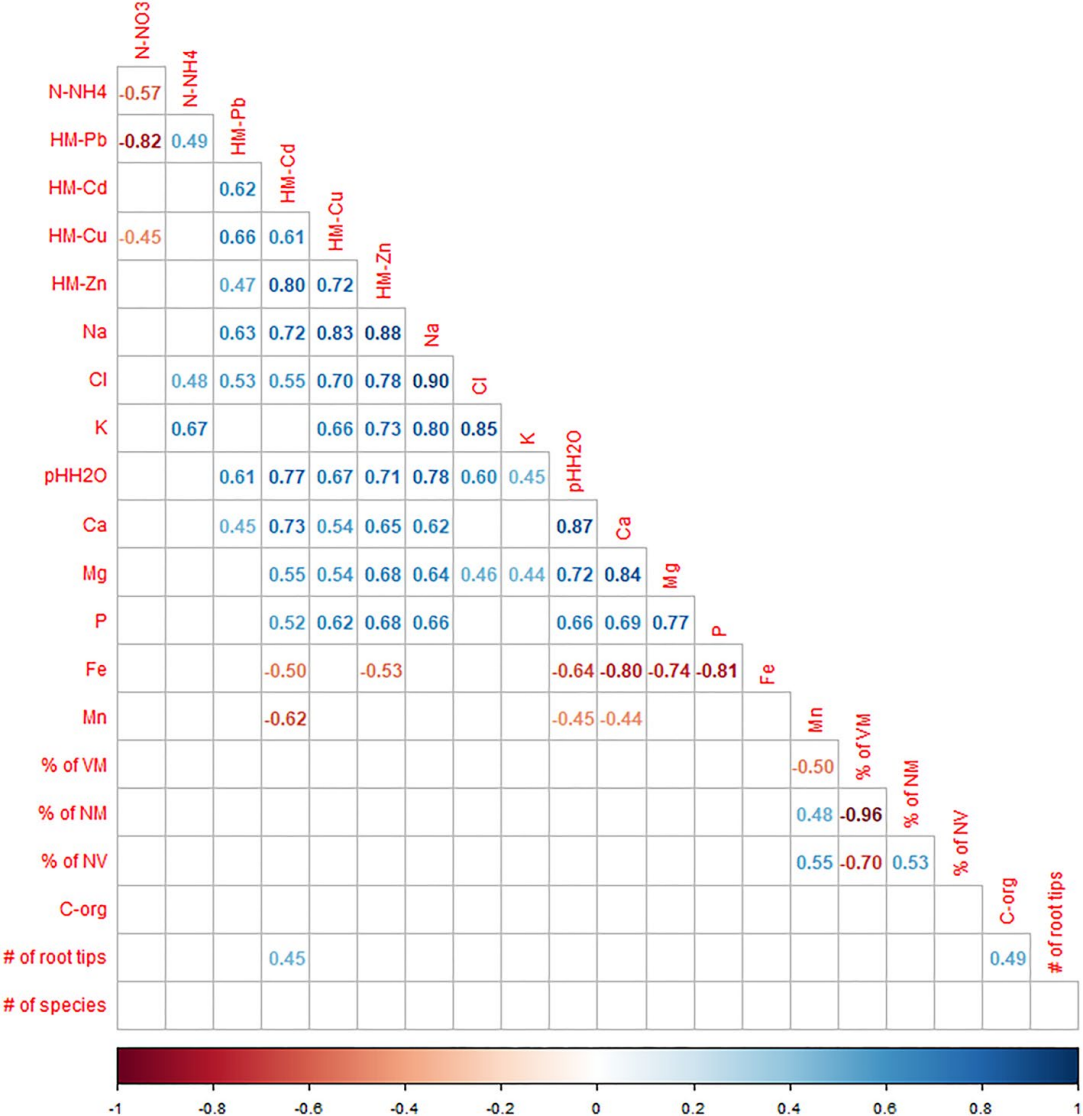
The significant, at *p* value < 0.05, Spearman correlations between the chemical and ECM characteristics of the 20 individual soil samples related to the trees examined in the study. The positive correlations given in blue and negative in orange to red colours. The strongest the correlation the darker font used, according to the color-legend.

The correlations, presented in Fig. 1, show moderate to strong positive relations between the content of heavy metals and components of road salt, i.e. Na and Cl. Additionally, the increase in the concentration of both of these element groups is related to the increase of the soil alkalinity and its Ca content. The Pb is the only heavy metal studied, which concentration is not related to any of commonly used soil fertiliser: K, P and Mg. However, the Pb concentration is positively correlated with the N-NH4 and negatively with the N-N03 abundance in the soil. The strongest negative relation, especially with P, Mg and Ca, was found for Fe. Therefore, the concentration of heavy metals, road salt components, Na, Cl, fertilisers K, P, Mg, soil alkalinity, Ca and Fe form a group of inter-related elements. The observed correlations varied from weak (0.45) to strong (0.90).

The weakest, negative relation with other chemical components was observed for Mn. The only soil element with no correlation with others was C_org_.

The strongest correlations between the root ECM characteristics were observed between the numbers of the ‘vital non-ECM’ (NM) and the ‘non-vital’ (NV) tips (strong negative relation, − 0.97); between NM and ‘vital ECM’ (VM) tips (moderate negative relation, − 0.70). All other correlations were weaker, ranging from 0.45 to 0.55. Positive correlations were observed between the VM and NV root tips abundance.

Finally, very few weak relations between the soil chemical and ECM characteristics were found (between 0.48 and 0.55). The soil content of C_org_ was positively correlated with the numbers of all root tips observed in the samples. Finally, Mn content was negatively correlated with the number of NM tips and positively with the numbers of NV and VM tips.

### Soil characteristics in the experimental sites

The analysis of the soil characteristics in two experimental sites, the urban and forest ones, based on ten soil samples from each site. The urban soil was alkaline with average pH equal to 7.61, whilst the forest soil was moderately acidic with mean pH of 5.53. According to the U Mann–Whitney test, the measured acidity was significantly different, see Table 1. No significant difference in the mean concentrations of the organic carbon, ammonium ion and Mn was observed. The nitrate ion and iron concentrations were higher in the forest samples. The concentration of K, Ca, Mg, Na, heavy metals Pb and Cd, Zn and Cu, were higher in the urban samples. The most abundant of all examined metals was Mg followed by Fe in the urban soil and Fe before Mg in the forest soil. The concentrations of the heavy metals (HM) decreased in the same way in both sets of samples: Pb > Zn > Cu > Cd.

**Table 1:** Soil chemical characteristics of study sites localized in the John III Sobieski Nature Reserve (forest) and Ursus district of Warsaw (urban).

Variable	U Mann–Whitney *p* value	Localization	
		Forest	Urban
Salinity			
C-org (%)	Ns^1^	2.0 ± 0.70	2.2 ± 0.35
Major nutrients			
N-NH_4_ (mg/l)	Ns	7.9 ± 3.4	9.0 ± 2.6
N-NO_3_ (mg/l)	0.028	37 ± 21	23 ± 5.1
P (mg/l)	0.00031	11 ± 8.5	40 ± 12
K^+^ (mg/l)	0.0058	45 ± 36	101 ± 37
Na^+^ (mg/l)	0.00030	25 ± 7.6	220 ± 233
Cl^−^ (mg/l)	0.0028	27 ± 7.8	85 ± 77
Mg^2+^ (mg/l)	0.001	46 ± 27	113 ± 32
Acidity			
Ca^2+^ (mg/l)	0.0015	731 ± 657	1750 ± 474
pHH_2_O	0.00021	5.5 ± 1.3	7.6 ± 0.61
Fe^2+^ (mg/l)	0.0052	191 ± 106	72 ± 53
Mn (mg/l)	Ns	20 ± 26	3.7 ± 1.7
Heavy metals			
Zn (mg/l)	0.00048	7.8 ± 7.5	31 ± 18
Cu (mg/l)	0.00018	1.2 ± 0.36	8.0 ± 3.87
Pb (mg/kg)	0.0091	24 ± 12	48 ± 34
Cd (mg/kg)	0.021	0.28 ± 0.24	0.58 ± 0.47

Means and standard deviations of selected chemical properties and pH of soil samples in each class of trees. Results of the U Mann–Whitney test for the differences between the concentrations of each of the chemical components.

*Ns* non-significant.

### Subdivision of the experimental sites based on soil characteristics

**Figure 2:**
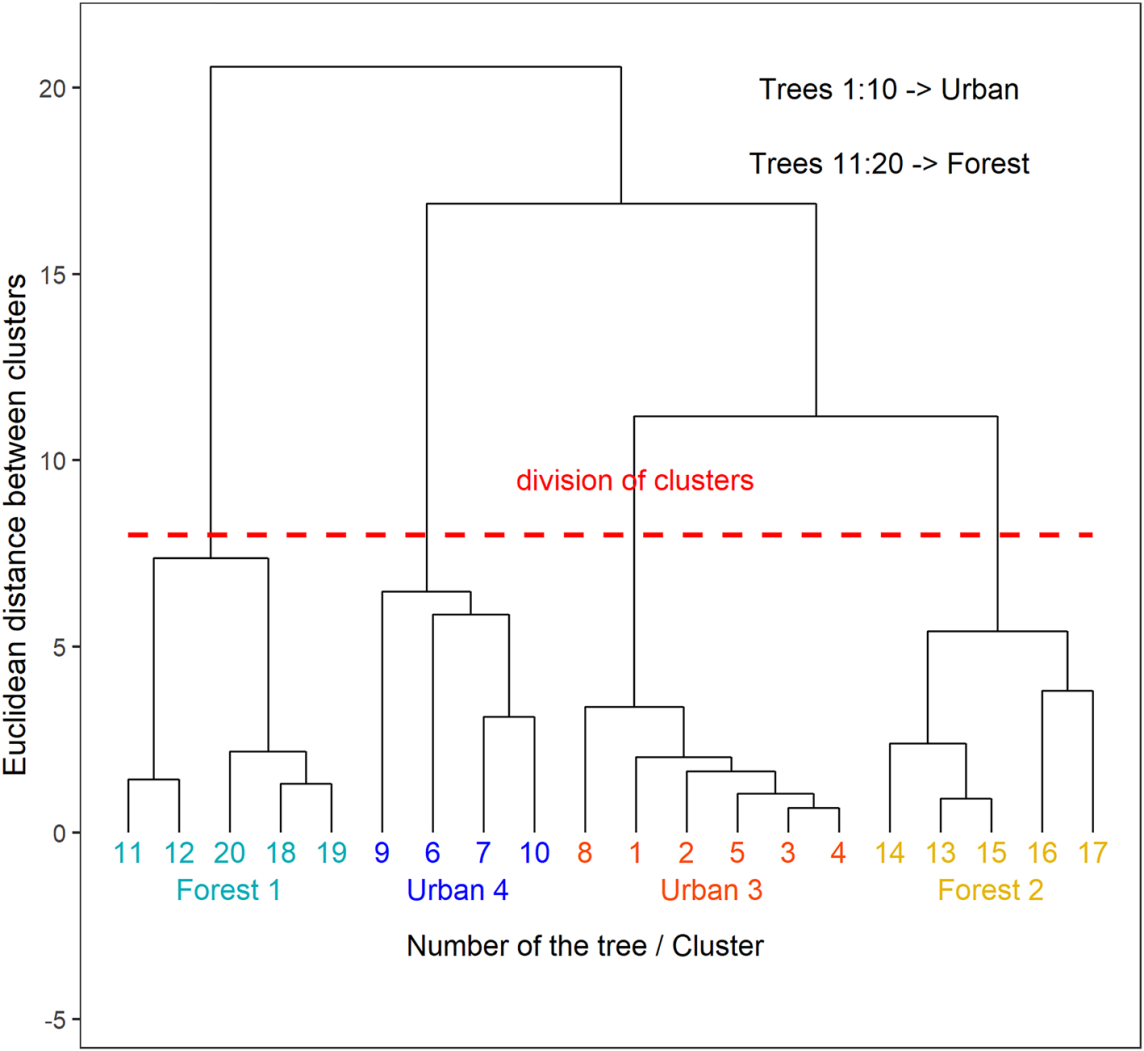
Dendrogram of the Agglomerative Hierarchical Clustering (AHC) of the 20 soil samples related to the examined silver birch trees. The AHC with Euclidean distance and Ward agglomeration method was used to identify clusters of trees which have grown under similar soil chemical characteristics. Trees with numbers 1 to 10 and numbers 11 to 20 were the urban and forest trees, respectively. The red dashed line indicates the division of the trees into 4 clusters.

It should be stressed that the characteristics of the urban and forest soil samples examined in this study show high variability between the samples, visible in high values of standard deviations in Table 1. Therefore, we examined their subdivision using clustering (the Agglomerative Hierarchical Clustering method) to obtain groups of samples of lower variability. Based on the dendrogram presented in Fig. 2, the examined trees were divided into four clusters: clusters 1 and 2 contained forest trees exclusively whilst clusters 3 and 4 contained urban trees exclusively. The differences between the soil samples from the four tree clusters are presented in Table 2. The homogenous groups of clusters based on the Kruskal–Wallis and the post-hoc Dunn’s tests are given for each soil attribute showing significant differences between the clusters.

**Table 2:** Chemical characteristics of clusters of soil samples introduced in the study.

Variable	Kruskal–Wallis *p* value	Tree clusters				Saline site
		Cluster forest 1	Cluster forest 2	Cluster urban 3	Cluster urban 4	Inowrocław-Mątwy^45^
Salinity						
C-org (%)	Ns^1^	1.7 ± 0.55	2.3 ± 0.76	2.2 ± 0.32	2.1 ± 0.42	
Major nutrients						
N-NH_4_ (mg/l)	Ns	9.2 ± 3.4	6.6 ± 3.1	7.3 ± 1.2	12 ± 2.1	
N-NO_3_ (mg/l)	0.011	21 ± 15 b	52 ± 14 a	24 ± 5.4 b	21 ± 4.7 b	
P (mg/l)	0.0016	5.0 ± 0.0 c	17 ± 8.3 bc	44 ± 12 a	34 ± 11 ab	
K^+^ (mg/l)	0.018	44 ± 24 b	46 ± 48 b	81 ± 19 ab	132 ± 39 a	34 ± 1.0
Na^+^ (mg/l)	0.0015	23 ± 3.4 b	27 ± 11 b	74 ± 48 ab	439 ± 230 a	280 ± 7.4
Cl^−^ (mg/l)	0.0065	26 ± 4.2 b	28 ± 11 b	38 ± 12 ab	154 ± 81 a	590 ± 12
Mg^2+^ (mg/l)	0.00081	24 ± 9.0 b	67 ± 20 ab	136 ± 9.3 a	78 ± 18 ab	14 ± 0.5
Acidity						
Ca^2+^ (mg/l)	0.0045	221 ± 96 b	1241 ± 560 ab	1877 ± 269 a	1561 ± 687 a	90 ± 3.5
pHH_2_O	0.0032	4.6 ± 0.63 b	6.5 ± 1.0 ab	7.5 ± 0.19 a	7.8 ± 1.0 a	
Fe^2+^ (mg/l)	0.0056	279 ± 50 a	102 ± 54 ab	56 ± 35 b	95 ± 72 ab	
Mn (mg/l)	Ns	36 ± 30	5.1 ± 6.8	4.3 ± 1.9	3.0 ± 1.2	
Heavy metals						
Zn (mg/l)	0.0015	2.9 ± 1.2 b	13 ± 8.2 ab	20 ± 3.9 a	47 ± 21 a	
Cu (mg/l)	0.0012	1.2 ± 0.39 b	1.1 ± 0.35 b	5.5 ± 1.6 a	12 ± 3.4 a	
Pb (mg/kg)	0.013	28 ± 15 ab	20 ± 7.9 b	33 ± 9.1 ab	70 ± 47 a	
Cd (mg/kg)	0.0067	0.15 ± 0.06 b	0.41 ± 0.29 ab	0.34 ± 0.18 ab	0.94 ± 0.57 a	
Geo-accumulation index						
Zn (I_geo_)	Not performed	− 4.6 ± 0.61	− 2.5 ± 0.94	− 1.8 ± 0.28	− 0.6 ± 0.64	
Cu (I_geo_)	Not performed	− 4.3 ± 0.46	− 4.5 ± 0.45	− 2.2 ± 0.41	− 1.1 ± 0.42	
Pb (I_geo_)	Not performed	− 0.093 ± 0.79	− 0.56 ± 0.56	0.14 ± 0.4	1.2 ± 0.96	
Cd (I_geo_)	Not performed	− 0.010 ± 0.61	1.5 ± 1.0	1.2 ± 0.79	2.7 ± 0.88	

Means and standard deviations of selected chemical properties and pH of soil samples within each cluster of trees. Results of the Kruskal–Wallis test for the differences between the concentrations of each of the chemical components with the homogenous groups of clusters. The geo-accumulation index values^85^ were computed for the HM in each cluster (the Kruskal–Wallis test was not performed in that case) in comparison to the geochemical background^46^.

*Ns* non-significant.

The division of the soil samples into clusters did not remove the strong variability of chemical characteristics within each cluster. The average numerical values of each chemical feature differed among the clusters, but the statistical differences given by the post-hoc test were negligible.

**Figure 3:**
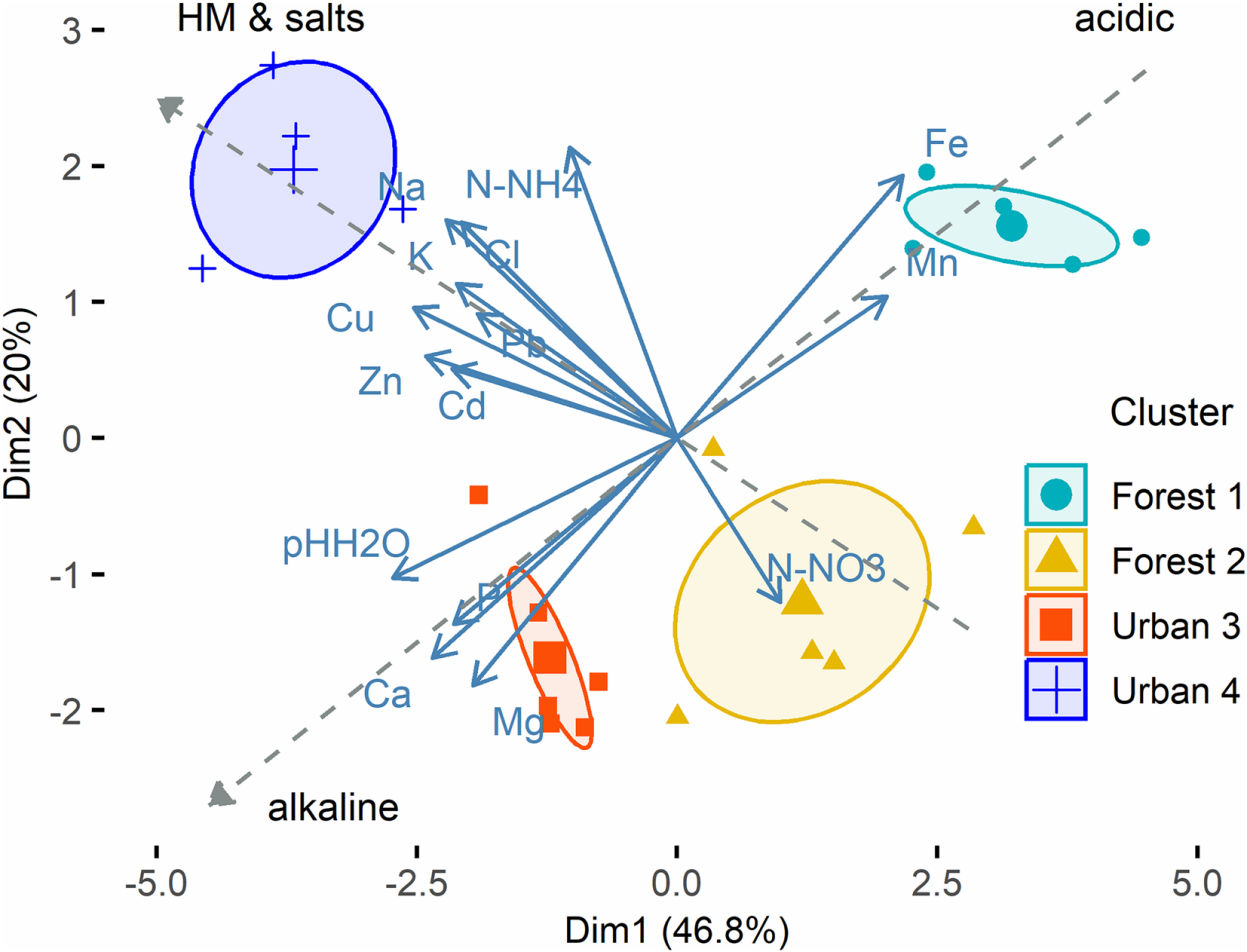
PCA plot representing the chemical characteristics of the soil samples related to the examined silver birch trees. The plot explains 66.8% of the total variance in all chemical characteristics of the 20 soil samples examined in the study. Each colour represents different cluster of samples, as in Fig. 1. The ellipses are the 95% probability confidence ellipses around the mean point of each cluster. The grey vectors indicate the gradients of the major changes in the chemical composition of the soil samples: change of the pH, from acidic to alkaline; increase concentration of HMs and salts.

Nevertheless, the differences between the samples belonging to different clusters are seen in the Principal Component Analysis (PCA) plot in Fig. 3. Firstly, the clusters differed along the gradient of the increasing pH and Ca content, increasing P and Mg and decreasing Fe concentrations. Forest cluster 1 had on average the lowest pH, the highest Fe and the lowest C_org_ concentrations. The direction of change led through the forest cluster 2 to the urban clusters. Secondly, the average concentrations of Na, Cl and HMs in both urban clusters were higher than in the forest ones but the two urban clusters differed from each other in the salinity level and heavy metals concentrations. The highest concentrations were found in the urban cluster 4.

The geo-accumulation indexes, were computed for the HM in the samples belonging to each cluster, see Table 2. The I_geo_ values confirm the results from the PCA analysis. The most contaminated urban cluster 4 was moderately polluted with Pb and moderately to strongly polluted with Cd (2 ≤ I_geo_ ≤ 3). The urban cluster 3 was also contaminated with Pb and Cd but at moderate level. Samples from the forest cluster 2 were only moderately contaminated with Cd and the samples belonging to the forest cluster 1 were not polluted with any of the examined heavy metals.

### ECM characteristics in the experimental sites

**Figure 4:**
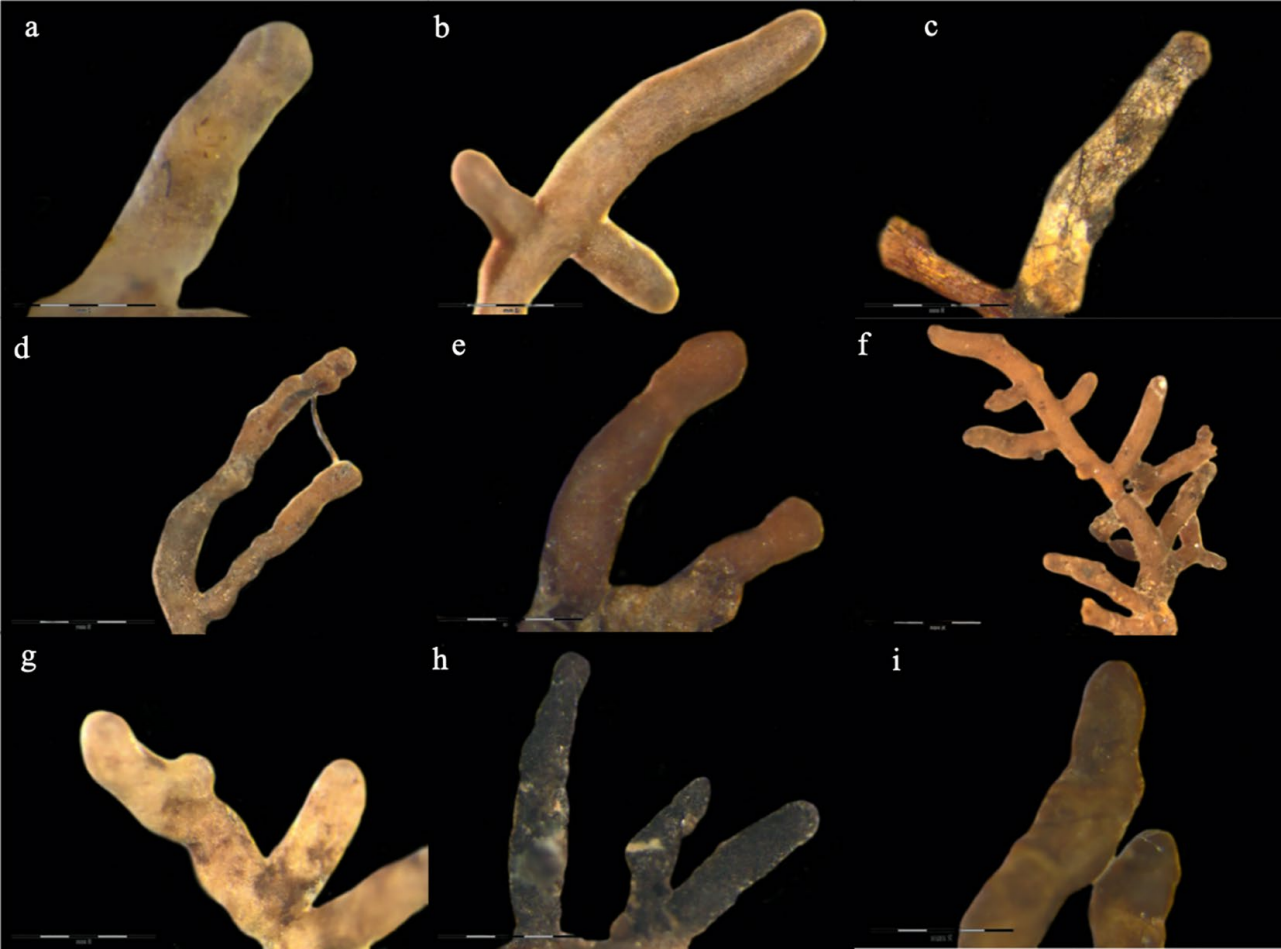
Morphotypes of ECM taxa of silver birch. (**a**) *C. bivelus*, (**b**) *G. hispidula*, (**c**) *L. quietus*, (**d**) *R. amoenolens*, (**e**) *R. exalbicans*, (**f**) *R. ochroleuca*, (**g**) *S. citrinum*, (**h**) *T. ellisii*, (**i**) *X. pruinatus*.

A total of eleven taxa were recorded for both study sites, with all of them being found in the forest location and nine in the urban site, see Table 3. Images of the nine, dominating ECM taxa are provided in Fig. 4. Only three taxa out of eleven belonged to Ascomycota: *Genea hispidula* Tul. & C. Tul., *Helotiales* sp., *Helotiaceae* sp.. Four and seven taxa, respectively, were present with more than 5% abundance in forest and urban sites: *Tomentella ellisii* (Sacc.) Jülich & Stalpers, *Russula exalbicans* (Pers.) Melzer & Zvára, *Russula amoenolens* Romagn., *G. hispidula* for forest site and *Scleroderma citrinum* Pers., *Cortinarius bivelus* (Fr.) Fr., *Russula ochroleuca* Fr., *T. ellisii*, *R. exalbicans*, *R. amoenolens* and *G. hispidula* for urban site. The forest samples were dominated by *T. ellisii*, 53%. Ectomycorrhizas by *R. amoenolens* and *R. ochroleuca* were more abundant in urban sites while those by *R. exalbicans* in forest sites (Table 3). As a result, the ECM species diversity was estimated using the Shannon index and was higher in the urban samples. The average diversity of the individual forest and urban samples was indifferent.

**Table 3:** Estimated occurrence of fungal taxa, root tips characteristics, species richness and diversity associated with the roots of silver birch trees, examined at the urban and forest sites (left columns), divided into 4 clusters as discussed in the study (right columns).

Identification	NCBI	Identity (%)	Forest		Urban		Forest 1		Forest 2		Urban 3		Urban 4	
			Freq	Abun	Freq	Abun	Freq	Abun	Freq	Abun	Freq	Abun	Freq	Abun
Basidiomycota														
*T. ellisii*	MZ773239	99	100	53.2	100	37.3	100	64.4	100	45.2	100	40.5	100	32.2
*L. quietus*	MZ773236	99	20	0.90	20	0.48	20	1.2	20	0.69	33	0.79	–	–
*S. citrinum*	MZ773238	100	60	6.0	30	7.4	60	3.3	60	7.9	17	0.58	50	18.2
*R. amoenolens*	MZ773233	99	70	8.8	60	17.4	60	5.7	80	11.0	67	16.8	50	18.5
*R. ochroleuca*	MZ773235	100	10	1.3	40	7.2	20	3.1	–	–	33	7.9	50	6.0
*R. exalbicans*	MZ773234	99	80	12.8	80	10.7	80	15.1	80	11.1	67	12.3	100	8.3
*C. bivelus*	MZ773232	100	10	3.3	20	6.0	–	–	20	5.7	17	0.44	25	14.8
*X. pruinatus*	MZ773240	100	10	1.2	10	0.63	20	2.9	–	–	17	1.0	–	–
Ascomycota														
*G. hispidula*	MZ773241	100	40	10.5	40	12.9	40	2.1	40	16.5	50	19.7	25	2.1
*Helotiales* sp.	MZ773237	99	10	1.2	–	–	–	–	20	2.1	–	–		
*Helotiaceae* sp.	MZ77324	98	10	0.90	–	–	20	2.1	–	–	–	–		
Number of		Total	Forest		Urban		Forest 1		Forest 2		Urban 3		Urban 4	
Trees		20	10		10		5		5		6		4	
Root tips		12,542	6018		6524		2576		3442		4032		2492	
NV root tips		280	88		192		63		25		134		58	
NM root tips		1648	910		738		412		498		471		267	
VM root tips		10,614	5020		5594		2101		2919		3427		2167	
Mean number of root tips per tree (mean ± standard deviation) Ns ^1^			602 ± 193		652 ± 78		515 ± 212		688 ± 141		672 ± 89		623 ± 57	
Fisher test of independence (*p* value < 0.001)^2^	Total mean													
NV (%)	2.2		***1.5↓***		***2.9↑***		2.4		***0.7↓***		***3.3↑***		2.3	
NM (%)	13.1		***15.1↑***		***11.3↓***		***16.0↑***		***14.5↑***		***11.7↓***		***10.7↓***	
VM (degree of mycorrhization) (%)	84.6		83.4		85.7		81.6		84.8		85.0		87.0	
Sum (%)	100		100		100		100		100		100		100	
Observed species richness														
Mycorrhizal fungal species [n]			11		9		9		8		9		7	
Chao-1			11		9		9		8		9		7	
Diversity														
Shannon diversity index (Hʹ)—for combined samples			1.58		1.78		1.27		1.62		1.58		1.73	
Shannon diversity index (Hʹ), mean of individual samples ± standard deviation	Ns^1^		0.95 ± 0.31		0.99 ± 0.30		0.89 ± 0.25		1.01 ± 0.38		0.98 ± 0.22		1.01 ± 0.44	

Data are the frequency (Freq.: percent of colonized plants) and abundance (Abun.; percent of mycorrhizal roots colonized) of fungal taxa on root tips based on the morphotype analysis. The contingency tables for the tree classes versus the abundance of the ‘vital ECM’ (VM) ‘non-vital’ (NV) or ‘vital non-ECM’ (NM) root tips^86^.

U Mann–Whitney test for comparison of urban versus forest means. Kruskal–Wallis test for comparison of cluster means.

Pearson residuals analysis: the values in the contingency tables in bold were significantly above or below the expected values (identified as those for which the absolute maximum of Pearson’s residual exceeded the value of 2). The upper (lower) arrows indicate values which were above (below) the expected values.

The Fisher’s exact test showed the distribution of the tip types in the classes of trees differed significantly from the average distribution. The percentage of vital ECM root tips (degree of mycorrhisation) was 83% and 86% for the forest and urban sites, respectively. On average, the urban class trees showed a significantly higher share of the NV type tips and lower content of the NM tips compared to the forest trees.

A more detailed division of the trees revealed that the soil samples related to urban trees from the most contaminated cluster 1 had the highest share of NV type tips. The lowest percentage of the NV type tips was observed among the samples from forest cluster 2. In comparison to the total average, samples from both urban clusters had decreased whilst both forest clusters had increased shares of the vital non-ECM (NM types) tips, respectively.

## Discussion

To the best of our knowledge, this is the first study that has examined the ECM community of *B. pendula* growing at an urban site. In the study, we directly addressed three of the anthropogenic stresses which may influence the ECM colonization. We measured the concentration of heavy metals Pb, Cd, Cu and Zn; salts, including the road salt elements Na and Cl; and acidity of the soil samples related to the examined ECM communities. All the above elements were positively correlated with each other. The correlations indicate that also the Fe, Mg and P contents were influenced by the anthropogenic transformation. A decrease of Fe content in parks versus urban soil was previously observed in Warsaw, Poland^11^. Compared with non-urban soils, higher contents of total P and P fractions in urban soil were observed along an urban–rural gradient in Nanchang, China^49^. As a whole, the above features describe an overall level of soil transition from natural to anthropogenically modified. A strong relationship between them made the task of separating the individual factors influencing the urban ECM community difficult.

No strong correlations were observed between the soil’s chemical features and the descriptors of ECM community characteristics, such as root tip vitality or exploration types, for the 20 individual tree samples. The results showed only a statistically significant, moderate, and positive correlation between organic carbon storage in soil and the total number of root tips. These results are in agreement with observations by previous studies^50–52^. Additionally, the share of the vital ECM (VM) root tips decreased and shares of the correlated ‘vital non-ECM’ (NM) and ‘non-vital’ (NV) increased with the rise of Mn content in the soil samples.

As no significant relationships were observed at an individual tree level, the focus in the study was on the comparison of the ECM characteristics in the groups of urban and forest soil samples. A very high variability in the chemical features was observed for both groups. Therefore, further clustering of the samples was performed. The four clusters were separated. The primary goal of limiting the variance in the clusters was not reached. However, according to the PCA analysis (Fig. 3), segmentation led to the separation of clusters of trees growing in the soil at different levels of anthropogenic transformation. In order to assess the severity of the transformation we made a serious of comparisons with other sites described in the literature.

First, we compared the average content of Na, K, Cl, Mg and Ca salts in the examined soil samples with the *B. pendula* site located near a soda ash factory at Inowrocław-Mątwy, central Poland^45^, see Table 2. The soil salinity at all four clusters was high as only Na and Cl concentrations were higher at the reference saline site, apart from the Na abundance at the urban cluster 4. The most striking effect of anthropogenic transformation is observed in the Ca concentrations in the analysed soil samples, which were from 2.5 (forest cluster 1) to over 90 (urban cluster 4) times higher than at the Inowrocław-Mątwy site.

Next, the observed pH and the HM content of the examined soil samples were compared with the pre-industrial background^46^, a control area of the silver birch growth in Kórnik, Poland^47^, urban soil in a small Polish city Grajewo characterized by high traffic^48^, Budapest, Hungary^3^ and a highly polluted by HM silver birch growth area in Bukowna, an industrial part of Silesia, Poland^47^, see Tables 2 and 4. As can be seen, only in the forest cluster 1 the mean pH was comparable with the control silver birch site, where it was 3.9. The soil of forest cluster 2 was of similar acidity as the contaminated soil in Silesia (with a neutral pH of 6.6). The soil samples collected in any of the urban areas were alkaline with a mean pH exceeding 7.3. Surprisingly, the mean HM concentrations in the examined soil samples turned out to be consistent with the levels observed in the natural environment in Kórnik^47^. The Cu, Zn and Cd levels were either lower (forest clusters) or comparable (urban clusters) with that natural habitat. The highest Cd content at urban cluster 2 was also lower than in small city Grajewo. Only the Pb concentration in urban samples (both clusters) was relatively high, but lower than in Budapest. As expected, the post-industrial control site at Bukowna showed much higher levels of extreme contamination with HMs than observed in Warsaw.

**Table 4:** The pH and the geo-accumulation index values^85^ computed in this study for the HMs in soils at four locations: a control area of the silver birch growth in Kórnik, Poland^47^, urban soil in a small Polish city Grajewo^48^, Budapest, Hungary^3^ and in Bukowna, a highly polluted by HM silver birch growth area in industrial part of Silesia, Poland^47^.

	Kórnik	Grajewo	Budapest	Bukowna
pHH_2_O	3.9 ± 0.0	7.8 ± 0.9	7.6 ± 0.3	6.6 ± 0.0
	Geo-accumulation index: I_geo_ = log_2_(C_n_/1.5B_n_) for heavy metals			
Pb	− 0.38 ± 0.079	− 0.51 ± 0.63	3.3 ± 0.50	6.3 ± 0.48
Cd	2.3 ± 0.65	3.5 ± 0.56	No data	9.7 ± 0.54
Cu	− 1.9 ± 0.18	− 1.9 ± 0.63	0.58 ± 0.59	0.96 ± 0.079
Zn	− 0.78 ± 0.22	No data	− 1.2 ± 0.50	6.6 ± 0.18

The geo-accumulation index values were computed in comparison to the geochemical background^46^.

Basing on the characteristics of the soil samples and the above comparison with other sites, we can describe the four clusters in the following way. Forest cluster 1, the natural forest habitat, most ferrous and acidic, with a pH similar to the natural area, and lowest HM concentrations. Forest cluster 2, the calcified forest habitat, more alkaline, with high Ca content, with the Cd concentration surpassing the background level, and low HM concentrations. Urban cluster 3, the moderately modified urban cluster, alkaline (as other urban sites), with increased HM concentration in comparison to the forest clusters, and moderately contaminated with Pb and Cd. Urban cluster 4, strongly modified urban cluster, the most contaminated of all the clusters, with the highest abundance of HMs.

Let us discuss the possible impact of individual sources of soil transformation on the ECM communities within the four clusters.

First, the average number of root tips was not significantly but numerically lower at the most natural forest cluster 1 than at all other clusters. The finding agrees with the positive correlation between the C_org_ content and the number of root tips made for the individual samples. The concentration of the organic matter in the natural forest cluster was the lowest, again but not significantly, of all sample groups.

Second, various studies have shown that a low pH (and P) level in the soil positively affects the formation of ectomycorrhizae^53–55^. The low pH favours the most natural forest cluster 1. Contrary to this, the ECM colonization level was high, above 80%, in all clusters and cluster 1 featured its lowest level (due to the significantly higher share of NM root tips). The highest colonisation was observed in the alkaline, most modified urban cluster 4. The observed phenomenon requires more in-depth study.

Third, Hrynkiewicz et al.^45^ investigated the effect of salinity on ECM colonisation of three tree species, *S. alba*, *S. caprea* and *B. pendula*, growing near a soda ash factory. A much lower level of *B. pendula* ECM colonisation, 30%, was observed than in the presented study. Nevertheless, *B. pendula* colonisation was stronger than for *S. alba* (15%) and *S. caprea* (9%), which may indicate high tolerance of silver birch to environmental stresses. Moreover, as observed by Bai et al.^56^ in the case of *Q. mongolica* seedlings, ectomycorrhizal inoculation can improve salt tolerance. Zwiazek et al.^57^, examining pine seedlings exposed to NaCl, also found reduced Na accumulation and higher growth rates in the case of seedlings inoculated with urban soil containing specific fungi genera. The Cl concentration at the most contaminated urban cluster 4 was much higher than at a forest level, and Na content was even higher than Hrynkiewicz et al.^45^ observed. The high colonisation in this cluster may be again related to relatively rich organic matter content. The concentration of C_org_ measured close to the soda factory was only 0.38% which may be related to high salinity decreasing soil organic carbon^58^.

Fourth, the clusters differed in average heavy metal soil content, but even in the most contaminated urban cluster 4, the soil samples were only moderately contaminated with Cd and Pb. Moreover, the observed level of contamination was several times lower compared to the post-industrial site in the vicinity of a non-ferrous metal smelter, heavily contaminated with heavy metals, investigated by Bierza et al.^47^. Therefore, the conclusions of Bierza et al.^47^ seem of limited application to the urban trees analysed in this study. The increased concentration of HMs in the urban soil did not lead to a decrease of ECM colonisation. This supports the findings of the Van Geel et al.^43^ study, which found that heavy metal pollution had little or no impact on ECM communities in urban areas.

Finally, the separate groups of trees differed insignificantly in species richness and diversity. The differences were very small, between seven and nine fungal species per cluster of soil samples, and some species were observed in minimal quantities. Therefore, there is no basis to generalize the presented results in this respect. Several studies targeting the diversity of ECM fungi in different taxonomic groups found no difference in species richness and diversity between heavily contaminated and non-contaminated soils^59,60^. Therefore, the observed lack of decrease in fungi taxa richness and diversity in the urban versus forest sites is understandable.

In summary, the fungal taxa in both urban and forest habitats were similar, and the study's hypothesis, stating that the urban ECM communities of silver birch are less developed than the forest ones, cannot be supported by the presented study. What then may account for the lack of differences?

Hui et al.^41^, who compared ECM communities in forests and urban parks, concluded that although ECM fungal richness was marginally greater in forests than in urban parks, urban parks still hosted rich and diverse ECM fungal communities. They suggested that the presence of host trees, rather than soil characteristics or even anthropogenic disturbance, determines ECM fungal community structure and diversity. The level of ectomycorrhizal colonization may indeed depend on the tree species. The study of ECM communities of pine seedlings by Zwiazek et al.^57^ found, contrary to this study, the lower abundance and diversity of ECM in seedlings colonized with the urban soil compared to seedlings growing in natural forest soil. On other hand, the research performed for *Tilia spp.*^37^, found a similar level of ECM colonisation of fine root trees growing in urban conditions (80%) as reported in this work.

Hence, one of possible explanations for the similarity of the *B. pendula* urban and forest ECM communities, may be the fact that *B. pendula* is a resistant tree species with tolerance to high pH, which allows for similarly high ECM colonisation at acidic and alkaline habitats^61^. The related *B. alleghaniensis* (*B. lutea)* and *B. papyrifera* species have been studied by Bainard et al.^38^. Their combined results for seven tree species with observed ectomycorrhizal colonisation showed significantly lower colonisation at the urban in comparison to the rural sites. However, the individual results for the *Betula* species were inconclusive. The ECM colonisation of the *B. lutea* roots was higher at the rural sites whilst the colonisation of *B. papyrifera* roots was higher at the urban sites. In both cases, the observed differences were not statistically significant. The lack of stress-related patterns of ECM colonisation level has been found for mountain birches (*B. pubescens ssp. czerepanovii*) by Ruotsalainen et al.^62^, investigating the ECM colonisation along three environmental gradients (two natural and one with human-induced pollution) within the Kola Peninsula (NW Russia).

Apart from the impact of a tree species, Tyburska et al.^40^ stated, after investigation of ECM colonization of white and black poplars, that “availability of water and nutrient and carbohydrates production and allocation, are the most important factor influencing the mycorrhizal colonization of urban trees.” A similar observation was made by Van Geel et al.^43^ after comparing the ectomycorrhizal fungal communities associated with *T. tomentosa* within and across urban areas in three European cities: “soil acidity, organic matter and moisture content were significantly associated with ECM community composition.” A strong correlation between the soil organic matter and ECM communities was also found by Jumpponen et al.^63^ in oak forests. It was also presented by Kennedy and Peay^64^, who showed that low soil moisture levels could decrease ECM colonization of the plants. The effect of the organic matter availability on *B. pendula* ECM colonization was also demonstrated in this study.

At the end of the study let us present a few observation concerning the individual fungi species. First of all, finding *Helotiales* sp. uniquely in forest trees (forest cluster 2) is unexpected. However, this species' very low frequency and abundance does not mean that they can be ascribed exclusively to forest habitats. *Helotiales* sp. is a large group of soil Ascomycetes with broad ecological niches from saprophytes to mycorrhizal symbionts. Moreover, they are groups of species highly adapted to saline conditions^45,65^.

Moreover, the presence of Russulaceae associated with *B. pendula* growing in the urban site studied had similar levels as found on protected sites, which may reflect a high HM resistance threshold of these fungi, supporting previous studies findings^39,47^. Disturbances, such as those caused by the elevated concentrations of HMs, may create a new niche in an environment, which in turn may accommodate certain species, such as *Russula* or *Scleroderma* species. These ectomycorrhizae are often found in disturbed habitats^66–68^. The most common fungal species from the family Thelephoraceae (e.g. *T. ellisii*) were shared between the taxa pools of studied communities and differed in both frequency and abundance. Ectomycorrhizae of *T. ellisii* and *Russula* spp. contribute to the group of contact exploration type^69^ and are often found in contaminated soils^70^ Hrynkiewicz et al.^45^ postulated that ectomycorrhizae of contact exploration type, having small absorption surface area, were able to significantly reduce heavy-metals uptake. The high abundance of mycorrhizae by *T. ellisii and Russula* spp*.* in urban site supports this view. High abundance of *S. citrinum* representing long-distance exploration ectomycorrhizae types in urban sites (Table 3) may be explained by their features. Long-distance ectomycorrhizae, due to large amounts of emanating hyphae, may explore and take up nutrients as well as heavy metals from a vast soil volume. This ability may allow metal–tolerant EMF species to act like natural filters preventing toxic metals transfer to the host tree and helping them with nutrient supply^28^. *Scleroderma* species are ubiquitous in temperate forests and their fruiting bodies are often found in disturbed habitats^66^, including mine tailings containing high concentrations of heavy metals^70^.

In conclusion, our study has shown no significant negative effect of the urban stresses on the ECM communities of *B. pendula*. The observation suggests that, regardless of their habitat differences, both forest and urban trees have the potential for generating a similar set of ECM taxa, even if in different proportions. The results might indicate a high tolerance of *B. pendula* to environmental stresses making that species a suitable plant material to establish on urban soils characterised by increased alkalinity and salinity, decreased Fe concentration and moderately contaminated with heavy metals. The examination of the individual trees showed that the organic matter concentration was a factor that affected the number of root tips.

## Methods

### Site description and sampling

**Figure 5:**
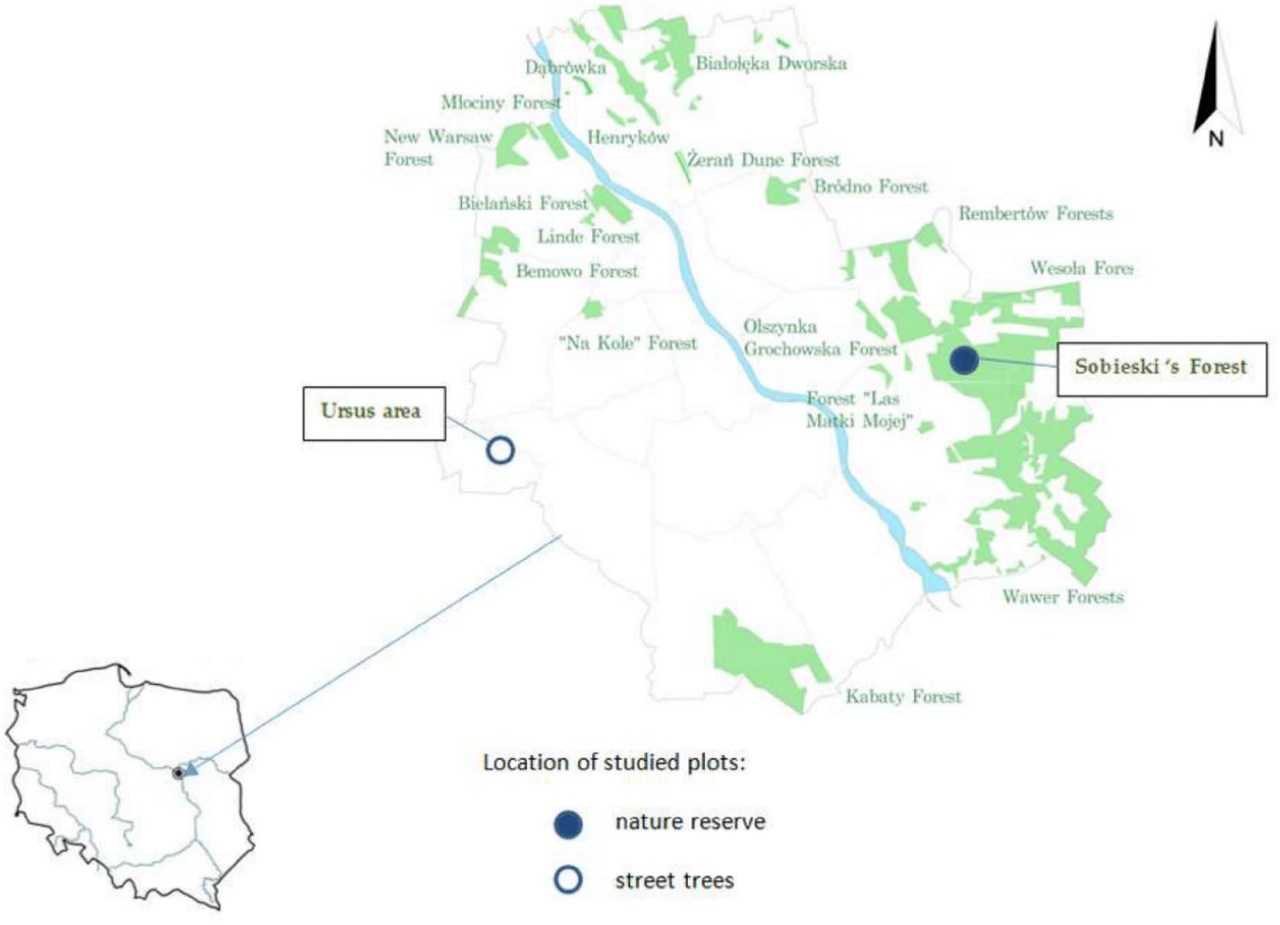
Location of studied trees on the map of Warsaw. The urban site was located in a post-industrial district of Warsaw, developed around Poland's largest factory of exhaust engines and tractors, Ursus SA. The control environment was the John III Sobieski Nature Reserve, officially protected since 1952, as a part of the Masovian Landscape Park. Figure prepared by the authors using Adobe Photoshop version 24.6^88^.

This study was conducted within Masovian Voivodeship in central-eastern Poland, see Fig. 5. The urban test site was located 15 km southwest of Warsaw city centre. The site with the highest degree of habitability was chosen for the study. It was located in a post-industrial district of Warsaw, which developed around Poland's largest manufacture of exhaust engines and tractors, Ursus SA., operating since 1923^71^. At present, it is the fourth most densely populated of 18 city districts in Poland and has a high level of road traffic^72^. In order to limit the impact of situational variables such as microclimate or weather conditions on the silver birch and its ECM communities, the control site was selected as close as possible to the test site. The control site, John III Sobieski Nature Reserve, is situated on the right bank of the Vistula River^73^ within the administrative borders of Warsaw. The John III Sobieski Nature Reserve has been officially protected since 1952 and is part of the Masovian Landscape Park. It provides a natural habitat where urban conditions on the ecosystem are limited.

Twenty *B. pendula* individuals were selected, ten trees per study site: urban and forest. The urban site was located alongside one street at intervals of approximately 6–7 m. Trees were randomly selected from the 5 m wide grass verge situated between the road and the pavement, i.e., growing in a limited unsealed, separated surface above the root zone, as illustrated in supplementary Fig_Suppl. 1. These trees had a high exposure to the road traffic and emissions, as well as the de-icing salts. The control site was located in the central part of the John III Sobieski Nature Reserve, in a 5 m wide strip of birchwood, between a woodland road. It was clear of vegetation other than birch trees in a clearing made for a power line to pass through, see supplementary Fig_Suppl. 1. Trees were randomly selected alongside the woodland road at intervals of approximately 6–7 m. The trees, selected for the study, in both locations were probably planted on each street at the same time, as an alley rows. *B. pendula* in both locations represented matured trees with diameter of the trunk between 42 and 60 cm. Trees were found in a good condition, without visible signs of dying. No dying branches have been observed, only a small number of dead twigs in a lower part of the crown, due to limited access to daylight.

Soil samples containing the feeding roots were collected from the urban and reference sites in May 2020. One sample per tree (a cube of soil with a side of 20 cm) was taken approximately 50 cm from the tree trunk. The samples were collected from a depth of 0–15 cm after removing grass and organic matter from the soil surface. Additional soil excavations were made between the trunk and sampling site, confirming that the samples taken contained roots of the surveyed trees only. The roots were traced directly from a tree stem. The soil-root samples were stored in sealed plastic bags at − 20 °C.

Simultaneously, 20 additional soil core samples (one per tree) were collected at the same distance from the tree trunk for chemical analysis. Soil samples (approximately 500 g each) were collected from a depth of 0–15 cm after removing any debris and stored in sealed plastic bags.

The collection of plant material performed in our study complies with relevant institutional, national, and international guidelines and legislation.

### Identification of mycorrhizae

Soil samples were defrosted in the lab at room temperature (ca. 23 °C for 30 min.), and soil particles were sieved from roots. The roots were washed gently under tap water to remove most of the soil debris^74,75^. Birch roots were separated from the roots of grasses and herbs. The separation of roots was based on the morphology (comparing with reference birch roots). The birch roots were classified according to Boratyński et al.^76^ keys and subsequently examined under a dissecting microscope at a 10–60× magnification. Mycorrhizas were classified into morphotypes (3–4 replicates represented each morphotype) based on macroscopic characteristics (colour, shape, texture, presence and organisation of the emanating hyphae and thickness of the mantle, rhizomorphs, and other elements) according to Agerer^77^. To confirm the identity of previously collected and preserved morphotypes, molecular analyses of two to three mycorrhizal tips of each unique morphotype were performed. The fungal ITS rDNA region was amplified with ITS1F/ITS4 primers^78,79^. Direct PCR was performed using the Phire Plant Direct PCR Kit (ThermoFisher Scientific, Waltham, MA, USA). To 10 μl of 2X Phire Plant PCR Buffer, 0.4 μl of Phire Hot Start II DNA Polymerase, 2.0 μl of 5.0 μM, each primer was added along with a mycorrhizal tip and additional H_2_O to reach 20 μl total volume. Cycling was performed using a Veriti 96-Well Thermal Cycler as follows: an initial denaturation step of 98 °C for 5 min, followed by 35 cycles of 94 °C for 5 s, 55 °C for 5 s and 72 °C for 20 s, and a final extension step at 72 °C for 5 min. Amplicons were visualised with UV illumination after Ethidium Bromide (Sigma-Aldrich, Milwaukee, WI, USA) staining. Excess dNTPs and unincorporated primers were removed from the PCR product using the Clean-Up Purification Kit (A&A Biotechnology, Gdynia, Poland). DNA was eluted in 40 μl H_2_O.

Sequencing PCR reactions were performed with 1 μl BigDye Terminator v. 3.1 Ready Reaction Mix (ThermoFisher Scientific, Waltham, MA, USA), 2 μl BigDye sequencing buffer (ThermoFisher Scientific, Waltham, MA, USA), 1 μl (5 μM) ITS4 primer and H_2_O to bring total volume to 10 μl. The thermal profile for sequencing reactions consisted of 25 cycles of 96 °C for 1 min, 96 °C for 10 s, 50 °C for 5 s and 60 °C for 105 s. The rDNA region was sequenced with an ABI 3500xl genetic analyzer (ThermoFisher Scientific, Waltham, MA, USA). For species identification, 97% alignment threshold over at least 450 base pairs was applied. Assignment of sequences obtained to ECM species was performed by BLASTSYSTEMS Identification Engine^80^.

### Physicochemical analysis of the soil

Samples of soil were air-dried, passed through a mesh screen, and stored for further analysis. The soil analyses were performed in the laboratory of the Polish Centre for Accreditation No. AB 312. For each soil sample, the pH was measured by mixing 40 ml of soil substrate with 100 ml of deionised water and 1 M KCl, respectively^81^. The exchangeable cations (Ca, Mg, K) were determined following the ISO 11,260^82^ protocol. The soil phosphorus (P) concentration was determined following extraction with 1% citric acid according to^83^. The total organic carbon (C_org_) was analysed using the Dumas method (PB 01 ed. 6, 20 05 2020) after complete oxidative combustion with a CHN-analyser LECO CHN-1000. Heavy metal concentrations of Cu, Zn, Pb and Cd in the soil were measured by digesting 40 mg of dried and sieved soil with 7.5 mL concentrated hydrochloric acid and 2.5 mL concentrated nitric acid^84^. Na, Cl, Fe, N-NNH4 and N-NN03 were determined using procedure PB: 07, 08, 09, 11, 13, 14, 18, 57 ed. 10, 20.05.2020.

### Data analysis

The geo-accumulation index (I_geo_) values introduced by Muller^85^ were calculated for the Cu, Zn, Pb and Cd to obtain an overview of the contamination levels in the examined sites. The geo-accumulation index was calculated according to the following formula:

$${\text{I}}_{{{\text{geo}}}} = {\text{ log}}_{{2}} \left( {{\text{C}}_{{\text{n}}} /{1}.{\text{5B}}_{{\text{n}}} } \right)$$

where C_n_ is the measured concentration of *n*th element in the soil, and B_n_ is the geochemical background for the *n*th element estimated as the pre-industrial background by Taylor and McLennan^46^.

The first analysis was performed to investigate the differences between the soil chemical characteristics of two classes of trees: urban trees versus forest trees. The differences in the characteristics of the urban and forest soil samples were examined with the non-parametric U Mann–Whitney test. The non-parametric test was applied due to the lack of normality of the examined features.

Next, the Agglomerative Hierarchical Clustering (AHC) with Euclidean distance and Ward agglomeration method was used to identify clusters of trees grown under similar soil chemical characteristics. The differences between the soil samples from each cluster of trees were studied in detail, separately for each soil attribute, with the non-parametric Kruskal–Wallis test. In the case of significant differences, the post-hoc Dunn’s test was used to identify the groups of samples with no indistinguishable values of the soil attribute.

The Principal Component Analysis (PCA), based on individual chemical characteristics of twenty examined trees, was carried out to graphically summarise the dispersion of the chemical features of the soil samples within and between the identified clusters.

The Spearman correlation was applied to examine the relationship between the soil's chemical characteristics and classes of root tips. The data was cross-tabulated into a contingency table and the Fisher’s exact test of independence was performed to understand the connection between tree classes and the abundance of ‘vital ECM’ (VM), ‘vital non-ECM’ (NM), and ‘non-vital’ (NV) root tips^86^. The cells in the contingency table were responsible for the significant departure from the independence of the examined variables were identified as those for which the absolute maximum of Pearson’s residual exceeded the value of two. The species diversity for each class of trees was estimated with the Chao1 and Shannon diversity indices.

The statistical analysis presented in this study were performed in the R program version 4.2.2^87^.

### Supplementary Information

Supplementary Information.

## Data Availability

The datasets generated during and/or analysed during the current study are available from the corresponding author upon reasonable request.
