# Supplementary material for: The impact of anthropogenic transformation of urban soils on ectomycorrhizal fungal communities associated with silver birch (Betula pendula Roth.) growth in natural versus urban soils
Source: Sci Rep. 2023 Dec 2;13:21268. doi: 10.1038/s41598-023-48592-6 (PMC10693619; doi:10.1038/s41598-023-48592-6)
Supplement: Supplementary file 1 — Supplementary Information. [file 41598_2023_48592_MOESM1_ESM.docx]

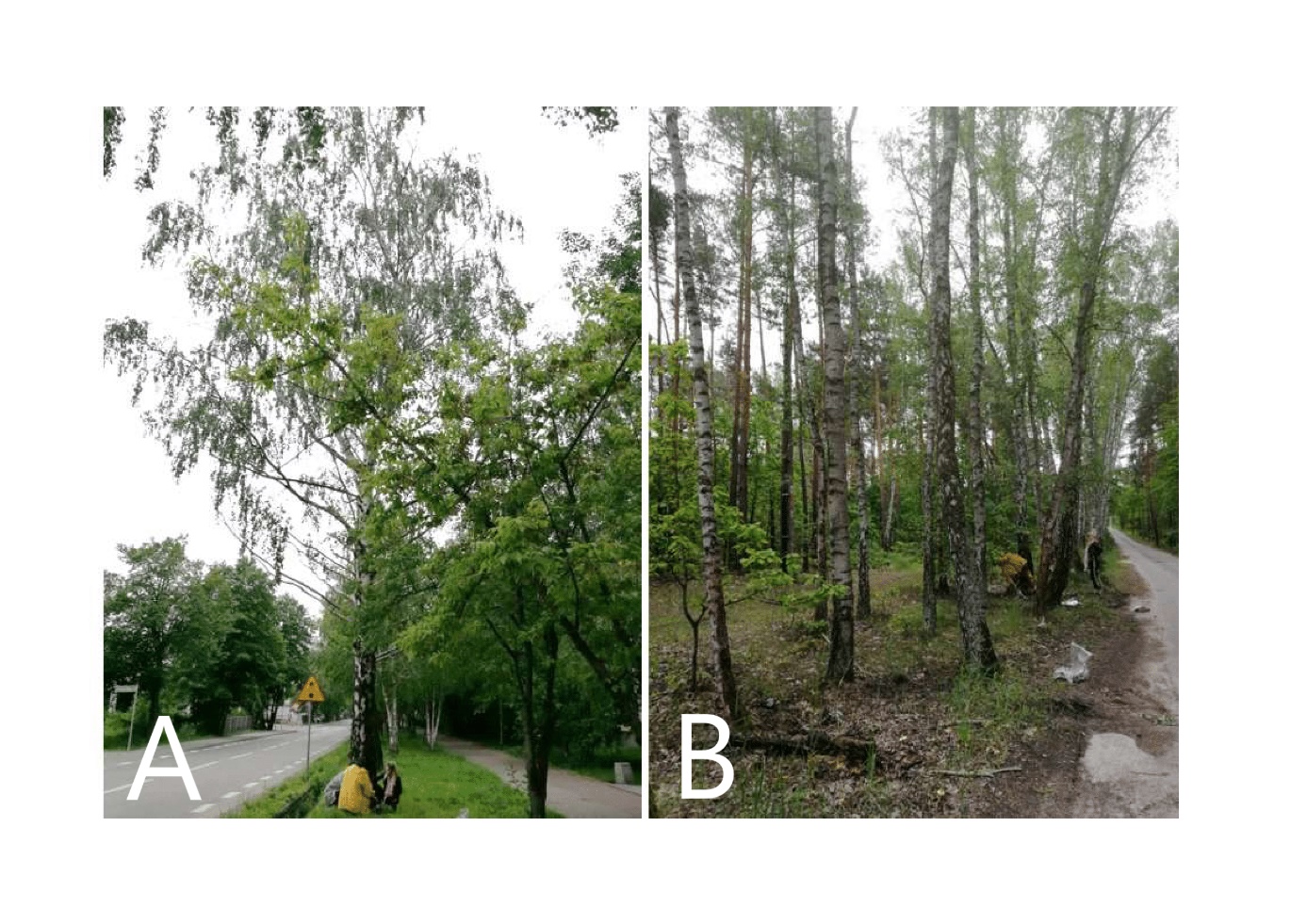


**Fig_Suppl. 1.** Experimental sites for the presented study of the *Betula pendula Roth.* ectomycorrhizal fungal communities. (A) Urban street trees in the Ursus district of Warsaw city, Poland. (B) Ground strip next to woodland road in the John III Sobieski Nature Reserve, within the Masovian Landscape Park, Poland. For detailed localization see map in **Fig. 5**, presented in the manuscript.
